# Potentiation Physiologically Proven

**Published:** 1893-06

**Authors:** Gustav Jaeger

**Affiliations:** Stuttgart, Germany


### POTENTIATION PHYSIOLOGICALLY PROVEN.

Prof. Dr. Gustav Jaeger, Stuttgart, Germany.

[Translated by B. Fincke, M. D.]

### Deduction for Bases and Acids.

From these results of the salts we may draw two conclusions, as I previously suggested: (1.) Regarding the difference of the bases of these various salts, therefore the degree of hostility to life of Natrum, Kali, and Caustic Ammonia. According to neural analysis, caustic Natrum and Ammonia are less hostile than Kali. Nobody will contend that this agrees perfectly with the practical experience with regard to Kali and Natrum. On the contrary, one might question whether the result of neural analysis concerning the difference of Kali and Ammonia corresponds with the experience by reference to the circumstance that the caustic action of Ammonia is much less than that of the caustic Kali. This objection, however, leads us directly to that difference in the action of matter frequently urged by me, viz., the difference between the *chemical* action, which increases with the concentration respectively with the mass, and the *neural* action, which reversely increases with the attenuation, therefore is an *action of the degree of volatility.* The latter is the object of neural analysis—*i. e.*, the *neural* action, and not the former, and on that point, that the caustic Ammonia has a much stronger *neural* action than the caustic Kali, there is no further arguing, for it is already conditioned by the difference of volatility; because the caustic Ammonia is much more volatile than caustic Kali, *ceteris paribus* its *neural* action must be much stronger, and so it is, indeed, in a high degree in both directions: (*a.*) In the action upon the *olfactory nerves.* Ammonia has a very strong, severe, and unpleasant odor, whilst caustic Kali acts weakly upon the olfactory nerves. (*b.*) Also, taken by inhalation, caustic Ammonia acts much stronger upon the whole organism than caustic Kali, producing cough, asthmatic sensations, etc. On this point I am very well informed by practical experience. Because caustic Ammonia is less caustic and destructive than the other caustics, Ammonia soap is preferred for the purpose of purifying woolen stuffs. On the contrary, the manufacture of Ammonia soap is much more fatiguing, noxious, and dangerous for the workingmen, on account of the exceedingly noxious and strong-acting Ammonia vapors, than the Natrum and Kali soaps, as I know from the workingmen themselves. Hence, also, in this point, experience and neural analysis agree perfectly.

(2.) A conclusion is permitted in the opposite direction, viz., from the measured low atomic nitric combinations upon the high atomic organic nitric substances, as the ptomaines, alkaloids, etc. The known poisonousness which such substances can unfold has its counterpart in the obstinacy with which the Ammonia-salts defend their poisonousness against the mode of attenuation. Also in this manner the neuro-analytical result can be defined as a correspondence with other experience.

The following table VI has no other numbers and substances as bases than the tables III–V. It only arranges them differently, and then adds new material in the column of the mean numbers, which enables us to draw a conclusion upon the second component of the measured salts. Whilst in the tables III–V the salts are arranged according to their *bases*, table VI arranges them according to the *acids* respectively, halogens, and the mean numbers permit now a conclusion upon the toxical properties of this factor. We could, of course, also submit these substances alone to neural analysis, but (1) this would be a new troublesome labor; (2) if we inhale an acid or halogen, we have a substance in which the *chemical* actions are much stronger than in the chemically indifferent salts, and the object of investigation in *potentiation*—for this is the only object of my work—is not the *chemical* but the *neural* action. For no difference of opinion is on the point that with the attenuation of a substance its chemical action decreases in the direct ratio to the mass, but only on that point the spirits are in the dark whether there is a kind of action in the substances which increases with their attenuation. This is concerned here; this is measured by neural analysis, and the more the chemical action can be kept in abeyance in this respect, the better it is.

Table VI gives us the following scale of poisonousness:

| | | | | |
|---|---|---|---|---|
| Chlorine-salts have the poison-number, | | | . . . . . . . . . . | 58 |
| Carbonic-salts | " | " | . . . . . . . . . . | 129 |
| Phosphoric-salts, | " | " | . . . . . . . . . . | 228 |
| Bromine-salts, | " | " | . . . . . . . . . . | 316 |

In considering these results we must keep the halogens apart from the genuine acids.

(*a.*) Halogens are two: *Chlorine*, with 58, and *Bromine*, with 316, as their poison-numbers. That the neural action of Bromine

### VI Table. The Lower Potencies of the Alkali-salts Arranged According to the Acids.

| | POTENCY. | 3 | 4 | 5 | 6 | 7 | 8 | 9 | 10 | 11 | 12 | 13 | 14 | 15 | 16 | SUM OF MINUS VALUES. |
|---|---|---|---|---|---|---|---|---|---|---|---|---|---|---|---|---|
| Brom-salts. | Na. | —35 | —34 | —30 | —22 | —16 | —22 | —13 | — 8 | + 4 | +11 | +18 | +16 | +14 | +19 | 180 |
| | Ka. | —70 | —45 | —33 | —31 | —26 | —32 | —26 | —25 | —28 | —19 | —17 | —13 | — 7 | + 5 | 372 |
| | Am. | —63 | —43 | —45 | —40 | —35 | —38 | —32 | —29 | —29 | —25 | —18 | —23 | —22 | —33 | 543 |
| | Mean. | —56 | —41 | —36 | —31 | —26 | —31 | —24 | —21 | —18 | —11 | — 6 | — 7 | — 5 | — 3 | 316 |
| Phosphor-salts. | Na. | —25 | —31 | —25 | —20 | —14 | —10 | + 1 | + 7 | +10 | +11 | +12 | +10 | | | 125 |
| | Ka. | —43 | —28 | —26 | —25 | —28 | —20 | —12 | —12 | —11 | — 8 | — 7 | — 2 | | | 222 |
| | Am. | —64 | —44 | —46 | —41 | —26 | —47 | —32 | —31 | —20 | —15 | —11 | —12 | | | 442 |
| | Mean. | —44 | —34 | —32 | —29 | —23 | —26 | —14 | —12 | — 7 | — 4 | — 2 | — 1 | | | 228 |
| Carbon-salts. | Na. | —14 | —16 | — 2 | + 5 | + 9 | +18 | | | | | | | | | 32 |
| | Ka. | —37 | —19 | —16 | —20 | — 8 | + 3 | | | | | | | | | 100 |
| | Am. | —50 | —36 | —36 | —24 | —37 | —21 | | | | | | | | | 223 |
| | Mean. | —34 | —24 | —16 | —13 | —12 | — 0 | | | | | | | | | 129 |
| Chlor-salts. | Na. | — 5 | + 6 | + 7 | +23 | +28 | | | | | | | | | | 5 |
| | Ka. | —40 | —23 | —13 | —14 | —10 | | | | | | | | | | 100 |
| | Am. | —34 | —22 | —25 | —30 | —23 | | | | | | | | | | 165 |
| | Mean. | —26 | —13 | —10 | — 7 | — 2 | | | | | | | | | | 58 |

surpasses that of Chlorine is incontestable: ($\alpha$) the odor of Bromine, which derives its name from *bromos*—stench—is by far more unpleasant, more hostile than that of Chlorine, and I still remember vividly an incident at my college time. An effort was made to cauterize a facial cancer with a Bromine paste. The infernal vapors of the Bromine drove the whole auditorium, and finally the operators out of the room. ($\beta$) Also, the dosage of the pharmacopœias agrees with it. In old Sobernheim the initial dose of Bromine water (1 : 40) is stated to be 5–6 drops, that of Chlorine water 1 scruple = 1.2 gram; therefore a gram reckoned as 20 drops, the fourfold quantity.

(*b*.) Of Acids we have two: Carbonic acid with 129, Phosphoric acid with 228, the latter, therefore, with a poison-number almost double. Undoubtedly, Phosphoric acid is much more poisonous than Carbonic acid, and it could only be wondered at that the difference in numbers is not still greater. In order to prove the cause of it, and whether the proportion is also the same with the free acids, of course further measurements are needed, which I omitted because they have no bearing upon the proper object of this work.

(*c*.) Now still remains the comparison of the halogens, on one hand, and of the acids on the other hand, and in this way:

($\alpha$) That Bromine-salts are more poisonous than Phosphoric acid is correct. According to Sobernheim, Phosphoric acid is "an odorless fluid of agreeable, sour taste, the mildest of the mineral acids," the initial dose 10 drops of the concentrated substance, whilst of Bromine the dose is only 5 drops of a solution of 1 Bromine to 40 water. ($\beta$) According to the table, Carbonic acid should be more hostile to life than *Chlorine*, if we may judge from the character of the salts directly upon that of their constituents. Now, this seems not permissible under all circumstances, and indeed not then when two such different salt-builders as Carbonic acid and Chlorine are concerned. Carbonic acid is known to be the weakest acid—so weak that in carbonic alkalies the caustic properties of the bases still utter themselves. Chlorine, on the contrary, is a very strong acid, and deprives its bases of the caustic properties entirely.

Probably in this the explanation of the neuro-analytical result is to be found, and especially in this way: In the Chlorine-salts the mutual connection and neutralization is more complete than in the carbonic acids, therefore they are more "neutral," less hostile to life than the carbonates, in the latter of which the greater poisonousness of alkaline substances still appears. The correctness of this explanation, of course, needs further provings, but here—*i. e.*, for our question—this is indifferent; on the other hand, the proper neuro-analytical result does not refer to the acids, but to the salts, and that kitchen salt is declared by neural analysis to be much less poisonous than Natrum agrees with other experiences in every respect; the first is the ideal of an indifferent, the latter of a lixivial salt.

### Conclusion.

This has to comprise several things:

1. The *method*. This is neural analysis. By its first exclusive application to the lower poisonous potencies of the substances treated, we obtained the possibility to prove the statements of this method upon their reliability by means of experiences with other methods. My desire was that this should have been done more extensively; but "all good things are three—not an infinite number." For anybody free of prejudice who is not yet bankrupt in the capacity for instruction, the stated arguments are sufficient—I will not say now to accept everything credulously, no, but in order to attain to the knowledge that we are not placed in the world to either believe or doubt, or to believe and to talk, but to act. Neural analysis has brilliantly stood the test in the field of poisonousness, and whoever has gained this view will also now look at the statements which we shall make in the next chapter in regard to the higher potencies and their animating effects with other eyes than if this preliminary proving had been omitted. Furthermore, I have in this relation to play out a trump—and indeed a *main trump*. It is an incontestable fact that all substances which have poisonous effects lose of these in violence by diminution of mass, respectively concentration, and that finally a point arrives where they cease altogether. Now let us look at the series of numbers of the seventeen measured salts whether they do not in all these seventeen substances likewise exactly express this fact. This correspondence, indeed, only refers to the final result; but if we examine the single series of numbers closer, we meet the same phenomenon which we already found in the measurements of Kali Carbonicum after swallowing: the decrease of the poisonousness with increasing attenuation forms *not a straight line,* we meet everywhere places where the line is broken, either so that in spite of the attenuation by one potency the poison-number has remained the same, or that it even has decreased; but the decrease is either greater or smaller than in the preceding and following potentiation. In regard to this peculiar course of the decrease of poisonousness in consequence of the attenuation we are indeed deficient in experiences in the toxicological or pharmacological department; but I ask: Has ever hitherto any prover of medicine, either allopathic or homœopathic, made such a finely graded systematic thorough proving of the various doses according to any method? According to my knowledge this has never been done, and, therefore, these fine differences have not yet been observed by anybody. This want of control of the method by other methods is however amply replaced inasmuch as not only *one* substance has been measured, but seventeen have been examined. If now a phenomenon repeats itself in every one of this seventeen series of measurements there is a guarantee given, that here the question is not of faults, accidents, and other things which can occur once and awhile, but of a process residing in the nature of things about which I have already expressed myself sufficiently when speaking of the potencies of Kali Carbonicum. The same is valid for the paralytic as well as for the animating action: their measure depends upon the energy of the molecular motion which is the product of mass and velocity. If in the attenuation the velocity of the molecules does not increase as rapidly as to cover not only the defect of the mass, but to overcompensate it, then not only the progress in the animating effect of the higher potencies ceases, but also the decrease in the paralytic effect of the lower potencies. This is as clear as anything can be.

2. In regard to the question of the dose, the result of the investigation is remarkable in the following directions:

*a*. It appears that it is quite erroneous to believe that in Homœopathy, in order to prevent initial poisonous effects which one wants to avoid, the gradual difference of poisonousness of the various substances could be neglected and the dosage treated according to a general pattern. In this respect it is certain, *First*, if poisonous effects are to be avoided, the attenuation must be carried at least to *indifference*.

*Second*, our investigation shows that the position of the point of indifference in general is a much higher one than the Homœopathy of to-day assumes. None of the seventeen salts is indifferent in the *third* trituration; in the so favorite 6th potency only 5 of the 17 have overstepped the point of indifference. Finally, if anybody thinks that already with the 15th potency he has done his whole duty he is still in error, for 3 of 17 salts produce in the 15th potency still paralytic phenomena, therefore poisonous effects—3 of 17 are 17 %.

*Third*, nobody, neither homœopath nor allopath nor myself, have expected that the position of the point of indifference shows so great differences, least of all, that within a relatively so nearly affined series of substances. Such varieties were possible as that between kitchen salt with indifference between the 3d and 4th potency, and Ammonium bromide with indifference in the 21st potency.

*b*. In view of these great differences, a *practical progress* in the question of dose demands at least a systematic thorough proving of the most common medicines in order to determine their points of indifference. Only then we know how high every one must be potentiated in order at least to be safe from poisonous effects. To be sure, it is not sufficient for this purpose that only one *single* person undertakes this proving; this is so clear that I here need not speak more about it. I only say: the patients who want to be cured homœopathically must be sure that they receive no medicines which are attenuated so insufficiently that they still produce poisonous effects in sensible persons. This justified demand is only to be fulfilled if for every substance the potency is determined in which it is at least indifferent in persons of great sensibility. I have proved that this can be accomplished by means of neural analysis. If anybody knows a better method well and good, let him come out.

3. In the above measurements the fact finds expression, that even among the Kali-salts, deemed harmless, there are such as even in such enormous attenuation as the billionth or trillionth, produce poisonous effects, paralytic symptoms, etc., when they are mixed with the respiratory air. This opens a remarkable insight into what we must denominate atmospheric poisons, and which, perhaps, is nothing else than what is called "genius epidemicus," or in the language of Paracelsus "sideric influence." I will hold back all the reflections, considerations, and observations here intruding upon me, because they would lead us too far away from the object and scope of the present work, but I did not want altogether to omit the hint in order to show also the reverse to those who are so well contented with their knowledge and power, viz.: how extraordinarily large the field is of which we know nothing, so that, therefore, they either have to investigate for themselves, or to learn from others who have taken pains to do it.

4. In conclusion, there is another point, in order to avoid a misunderstanding. The word "indifference" is capable of a different interpretation, and therefore it must here be said what I understand by it, and this of course is nothing else but what I have expressed in numbers. A potency which in the neuro-analytical proving produces neither paralytic nor animating effects, I call with the same right indifferent, as the solution of kitchen salt is called indifferent, which in living tissues, *i. e.*—blood-corpuscles—produces neither swelling nor shrinking, or as a temperature in relation to another body is called indifferent if at the latter it produces neither an increase nor a decrease of its heat. What, therefore, with "indifference" is not intended to be said is that the injection of a substance in a dose denominated by me "indifferent" be *perfectly* harmless in a living being. Besides other things of which I shall speak later on, this cannot be the case, because secretion or combination change more or less rapidly the degree of concentration upon which depends the indifference.

[TO BE CONTINUED.]

